# Modulation of Plant-Derived Bioactive Phenolic Compounds by Cytokinins in *Hypericum amblysepalum* Shoot Cultures

**DOI:** 10.3390/plants15071017

**Published:** 2026-03-26

**Authors:** Hilal Surmuş Asan

**Affiliations:** Department of Biology, Faculty of Science, Dicle University, 21280 Diyarbakır, Turkey; hilalsuran@gmail.com

**Keywords:** *Hypericum amblysepalum*, cytokinin, shoot culture, plant-derived bioactive compounds, LC–MS/MS, phenolic compounds, antioxidant activity

## Abstract

Cytokinins are key plant growth regulators that play an important role not only in morphogenesis but also in the regulation of plant-derived bioactive compound production in plant tissue culture systems. In this study, the effects of two cytokinins, 6-benzylaminopurine (BAP) and zeatin (ZEA), applied at four concentrations (0.1, 0.25, 0.5, and 1.0 mg L^−1^), were compared in terms of biomass production and phenolic compound accumulation in shoot cultures of *Hypericum amblysepalum* Hochst. Both cytokinins significantly enhanced plant growth compared to the control, with the highest dry weight (26.5 ± 8.6 mg DW) and shoot number (11.2 ± 4.4 shoots per explant) recorded in cultures supplemented with 0.1 mg L^−1^ BAP. In contrast, ZEA was more effective in stimulating the accumulation of secondary metabolites. LC–MS/MS analysis revealed that 0.1 mg L^−1^ ZEA markedly increased the accumulation of major plant-derived bioactive phenolic compounds, including hypericin, pseudohypericin, chlorogenic acid, rutin, luteolin, hesperidin, luteolin-7-glucoside, and hyperoside, compared to the control. Consistent with these findings, total phenolic content (TPC) and total flavonoid content (TFC) were significantly higher in ZEA-treated cultures, which also exhibited stronger DPPH radical scavenging activity, indicating enhanced antioxidant potential. Overall, these results demonstrate that BAP is more suitable for biomass enhancement, whereas ZEA is more effective in improving the production of bioactive phenolic compounds and antioxidant capacity in *H. amblysepalum* shoot cultures, highlighting their potential as a sustainable source of valuable plant-derived bioactive metabolites for pharmaceutical applications.

## 1. Introduction

The genus *Hypericum* L., a member of the *Hypericaceae* family, comprises small trees, herbs, and shrubs and includes approximately 484 species distributed across diverse ecosystems worldwide [1]. In the flora of Turkey, the genus is represented by nearly 96 species and two subspecies, of which 45 taxa are endemic [2]. Species of *Hypericum* have long been used in traditional medicine for the treatment of bruises, burns, inflammation, swelling, anxiety, bacterial, and viral infections, as well as for their antidepressant and mild sedative effects [3,4].

*Hypericum* species are characterized by a rich phenolic profile, including hypericin, pseudohypericin, quercetin, hyperforin, hyperoside, adhyperforin, biapigenin, and quercitrin [5]. In recent years, increasing attention has been paid to the characterization of plant-derived bioactive compounds from *Hypericum* species, as these metabolites exhibit a wide range of pharmacological activities, such as antidepressant, antibacterial, anti-inflammatory, antiviral, neuroprotective, antinociceptive, and anticancer effects [6,7]. Due to these biological activities, *Hypericum* species have become important targets for phytochemical and biotechnological studies aimed at increasing the production of valuable secondary metabolites.

Cytokinins are widely used in shoot culture systems due to their ability to promote cell division, stimulate axillary shoot proliferation, and regulate organogenesis under in vitro conditions [8]. Unlike auxins, which are primarily associated with root induction and callus formation, cytokinins play a central role in shoot biomass enhancement, making them particularly suitable for studies aiming at shoot-based metabolite production. In addition, cytokinins have been reported to stimulate phenolic compound accumulation by activating the phenylpropanoid pathway and enhancing antioxidant defense mechanisms. These effects are often amplified in plant tissue culture systems, where hormonal responses are more pronounced [9]. In vitro culture systems provide valuable tools for studying metabolite biosynthesis and for enhancing the yield of bioactive compounds compared to field-grown plants [10]. Numerous *Hypericum* species have successfully been propagated and studied using in vitro culture techniques [11,12,13].

*Hypericum amblysepalum* is a regionally distributed species, occurring mainly in South and Southeastern Anatolia in Turkey, with additional populations in northern Iraq and the Syrian desert [14]. Despite its local importance, scientific information on this species remains limited compared to other members of the genus. Previous studies have demonstrated that extracts from flowers, fruits, and seeds possess significant antioxidant, anticancer, and anticholinesterase activities, including promising cytotoxic effects against human cervical cancer cells, indicating its pharmacological potential [15]. However, investigations focusing on in vitro culture systems, biomass optimization, and controlled enhancement of secondary metabolite production in this species remain scarce. Considering its restricted distribution, proven bioactivity, and the lack of comprehensive in vitro studies, *H. amblysepalum* represents an underexplored yet valuable candidate for phytochemical research and sustainable utilization of regional plant genetic resources.

Therefore, the aim of this study is to evaluate the effect of different cytokinin types and concentrations on shoot proliferation and phenolic compound production in *H. amblysepalum* shoot cultures, with the objective of contributing to the development of sustainable biotechnological strategies for the production of valuable plant-derived bioactive compounds.

## 2. Results

Plantlets cultured in the control (plant growth regulator (PGR)-free) medium developed as single shoots with thick stems and broad, dark green leaves. Murashige and Skoog (MS) medium supplemented with 6-benzylaminopurine (BAP) substantially increased shoot dry weight compared to the control (5.27 ± 0.35 g DW per explant). The greatest mean dry weight (26.5 ± 2.15 g DW per explant) was observed at 0.1 mg L^−1^ BAP, with similar values across all BAP treatments (0.1–1.0 mg L^−1^). BAP also promoted greater shoot proliferation than zeatin (ZEA), with the highest average shoot number per explant recorded at 0.1 mg L^−1^ BAP (11.29 ± 4.48). Shoot elongation was reduced in all PGR-treated groups compared to the control (4.8 ± 0.78 cm), and among ZEA treatments, the highest shoot length (3.4 ± 0.75 cm at 0.5 mg L^−1^) exceeded that of all BAP treatments (average 1.03–2.1 cm); however, no significant differences were detected among ZEA concentrations (Table 1). The plants grown on PGR-supplemented media maintained healthy morphology with large, green leaves (Figure S1).

### 2.1. LC–MS/MS Phenolic Profile

Hyperoside, rutin, and pseudohypericin were identified as the most abundant constituents through LC–MS/MS analysis. Furthermore, apigetrin was not detected, whereas luteolin, apigenin, luteolin-7-glucoside, and hyperforin were the least abundant compounds (Table 2).

The contents of the naphthodianthrones hypericin (52.60 µg g^−1^) and pseudohypericin (169.90 µg g^−1^) were highest under 0.1 mg L^−1^ ZEA treatment, substantially exceeding the levels observed in control and BAP treatments. Hyperforin was undetectable in control and BAP-treated cultures but was present at low levels in ZEA-treated samples, particularly at 0.1 and 1.0 mg L^−1^. Flavonoid content generally increased under 0.1 mg L^−1^ ZEA treatment, with rutin and hyperoside showing notable elevation (261.50 and 3744.9 µg g^−1^, respectively). Quercetin peaked under 0.5 mg L^−1^ BAP (51.10 µg g^−1^), indicating that higher BAP concentrations may favor accumulation of certain flavonoids. Quercitrin was highest in control samples (128.00 µg g^−1^) and decreased with cytokinin treatments. Other flavonoids exhibited moderate fluctuations, with some showing slight increases under ZEA or specific BAP doses. Overall, 0.1 mg L^−1^ ZEA promoted accumulation of several flavonoids more effectively than BAP, though certain flavonoids favored higher BAP concentrations. Regarding phenolic acids, protocatechuic acid concentration was highest at 0.5 mg L^−1^ BAP (20.35 µg g^−1^), with moderate increases also seen at 0.1 and 1.0 mg L^−1^ ZEA. Chlorogenic acid content peaked under 0.1 mg L^−1^ ZEA (66.30 µg g^−1^) and 0.5 mg L^−1^ BAP (50.80 µg g^−1^), indicating both cytokinins at specific concentrations can enhance phenolic acid accumulation (Table 2).

These results demonstrate that phenolic acid synthesis responds positively to both cytokinins, with specific optimal concentrations.

### 2.2. Total Phenolic Content (TPC)

The total phenolic content (TPC) of *H. amblysepalum* shoot cultures was significantly influenced by cytokinin treatments (Table 3). PGR-free cultures exhibited the highest phenolic accumulation (18.11 ± 0.58 mg GAE g^−1^ DW), showing a marked increase compared to all cytokinin-treated groups.

Among cytokinin applications, ZEA treatments generally resulted in higher TPC values than BAP treatments. The highest TPC among ZEA-treated cultures was observed at 0.1 ZEA (12.48 ± 0.46 mg GAE g^−1^ DW), followed by 0.5 ZEA (11.79 ± 1.14 mg GAE g^−1^ DW) and 0.25 ZEA (10.59 ± 1.29 mg GAE g^−1^ DW). In contrast, BAP treatments led to moderate phenolic accumulation, with TPC values ranging from 6.47 ± 1.02 to 9.31 ± 0.66 mg GAE g^−1^ DW, depending on the concentration applied.

### 2.3. Total Flavonoid Content (TFC)

The total flavonoid content (TFC) of *H. amblysepalum* shoot cultures varied significantly depending on the applied cytokinin treatments (Table 3). PGR-free cultures showed the highest TFC value, reaching 43.79 ± 0.98 mg QE g^−1^ DW.

Among cytokinin treatments, ZEA applications resulted in markedly higher flavonoid accumulation compared to BAP treatments. The highest TFC among ZEA-treated cultures was observed at 0.1 ZEA (38.37 ± 0.58 mg QE g^−1^ DW), followed by 0.5 ZEA (37.13 ± 1.11 mg QE g^−1^ DW) and 0.25 ZEA (35.76 ± 2.62 mg QE g^−1^ DW). In contrast, BAP treatments led to relatively lower flavonoid levels, with TFC values ranging from 13.27 ± 1.16 to 21.30 ± 2.13 mg QE g^−1^ DW across the tested concentrations.

### 2.4. DPPH Radical Scavenging Activity

The DPPH radical scavenging activity of *H. amblysepalum* shoot cultures showed no notable variation among the different cytokinin treatments (Table 3). PGR-free cultures exhibited antioxidant activity of 18.2 mg ASA g^−1^ DW, while cytokinin-treated cultures ranged from 17.2 to 18.65 mg ASA g^−1^ DW. The highest value was observed in cultures treated with 1.0 mg L^−1^ ZEA (18.65 ± 0.010 mg ASA g^−1^ DW), although all treatments showed comparable activity.

## 3. Discussion

Cytokinin-induced modifications in morphology and secondary metabolite profiles have been widely reported in *Hypericum* shoot proliferation systems [16,17,18]. In the present study, all tested cytokinins significantly influenced cell growth and differentiation in *H. amblysepalum* shoot cultures. While control plantlets developed as single shoots, cytokinin supplementation markedly enhanced shoot proliferation, confirming the essential role of exogenous plant growth regulators in in vitro shoot induction. Similar stimulatory effects of cytokinins on shoot proliferation have been reported in other *Hypericum* species, where BAP supplementation significantly enhanced shoot multiplication and regeneration in *Hypericum perforatum* and *Hypericum spectabile*, demonstrating the promotive role of cytokinins on in vitro shoot development [19,20].

Among the tested cytokinins, BAP exhibited the strongest effect on shoot multiplication and biomass accumulation. This finding is consistent with previous reports describing BAP as the most effective cytokinin for shoot regeneration in several *Hypericum* species [12,17,18]. In the present study, 0.1 mg L^−1^ BAP was sufficient to promote multiple shoot formation while maintaining optimal shoot length and plantlet vigor. Higher BAP concentrations have been reported to induce growth abnormalities, such as dense leaf formation and basal tissue darkening, in various *Hypericum* taxa, highlighting the importance of cytokinin optimization [18,19,20,21].

In contrast, ZEA supplementation resulted in lower shoot numbers but significantly increased shoot elongation, a response previously observed in other medicinal plants and attributed to the distinct physiological role of ZEA in cell elongation and differentiation [22,23]. Similar genotype-specific responses to cytokinins were reported in *H. perforatum*, with increased biomass observed under BAP and less favorable effects under zeatin [16].

Beyond morphological effects, cytokinins exerted a pronounced influence on phenolic metabolism [11,16,17]. LC–MS/MS analysis revealed that hyperoside, rutin, and pseudohypericin were the dominant phenolic compounds across all treatments, whereas hyperforin and certain flavonoids were present at comparatively low levels, consistent with previous reports in *Hypericum* shoot cultures [18]. Notably, shoots cultured with 0.1 mg L^−1^ ZEA exhibited the highest accumulation of major plant-derived bioactive phenolic compounds, including hypericin, pseudohypericin, chlorogenic acid, rutin, and luteolin derivatives. Similar ZEA-mediated stimulation of hypericin production has been reported in *H. sampsonii* and *H. perforatum* shoot cultures [11,12,13,14,15,16].

Our findings demonstrated that although BAP treatment enhanced the accumulation of several phenolic compounds, including hypericin, pseudohypericin, rutin, and quercetin, the magnitude of this increase was lower than that observed under ZEA treatment. This is consistent with previous studies showing that the type of cytokinin influences phenolic compound accumulation, with differential effects observed among BAP, zeatin, and other cytokinins in plant tissue cultures [24]. Such cytokinin-specific regulation of secondary metabolism has been associated with differential activation of the phenylpropanoid pathway and related biosynthetic enzymes [25].

Variations in total phenolic content (TPC) and total flavonoid content (TFC) among the experimental groups were generally consistent with the qualitative and quantitative changes detected by LC–MS/MS analysis. Treatments exhibiting elevated TPC and TFC values were characterized by a coordinated increase in multiple phenolic acids and flavonoids rather than the accumulation of a single dominant metabolite. It should be emphasized that TPC and TFC represent integrated spectrophotometric responses reflecting overall reducing capacity rather than the absolute sum of individual compounds quantified by LC–MS/MS. Nevertheless, the parallel trends observed across these analytical approaches indicate a robust cytokinin-mediated modulation of phenolic metabolism in *H. amblysepalum*. Similar concordance between spectrophotometric total phenolic/flavonoid measurements and detailed chromatographic phenolic profiles has also been reported in various *Hypericum* species, where phenolic accumulation patterns across plant organs and culture conditions were found to correlate with LC-based phenolic profiling [26,27].

Antioxidant activity, as assessed by DPPH radical scavenging capacity, tended to increase with both TPC and TFC values. Cultures exhibiting higher phenolic and flavonoid contents consistently demonstrated enhanced antioxidant potential, supporting the central role of these metabolites in free radical scavenging activity. The elevated DPPH inhibition observed in ZEA-treated cultures can be attributed not only to increased total phenolic levels but also to qualitative changes in phenolic composition, as compounds such as hypericin, chlorogenic acid, quercetin, luteolin, and their glycosides possess high radical scavenging efficiency [16].

Overall, these findings demonstrate that cytokinin type plays a decisive role in regulating both biomass production and bioactive phenolic compound accumulation in *H. amblysepalum* shoot cultures, in agreement with previous reports highlighting the regulatory role of cytokinins in plant growth and secondary metabolite biosynthesis [12,13,16]. While BAP appears more suitable for enhancing shoot proliferation and biomass production, ZEA is particularly effective in promoting the accumulation of valuable plant-derived bioactive compounds and antioxidant capacity. Similarly, our previous study showed that elicitor treatments significantly modulated secondary metabolite accumulation in *H. amblysepalum* cultures [28]. These findings collectively demonstrate that in vitro metabolic responses are highly dependent on the type of regulatory factor applied. Such cytokinin-dependent modulation further highlights the potential of *H. amblysepalum* shoot cultures as a sustainable biotechnological platform for the production of high-value bioactive metabolites.

## 4. Material and Methods

### 4.1. Plant Material

The seeds of *H. amblysepalum* were gathered from mature wild plants in the Bakırkırı vicinity of Mardin. Voucher specimens No: DUF-211 are deposited in the Herbarium of the Department of Biology, Faculty of Sciences, Dicle University, Diyarbakır (Turkey).

### 4.2. Chemicals

Standart compounds of hypericin (≥95%), pseudohypericin (≥95%), hyperforin (≥98%), hyperoside (≥97%), chlorogenic acid (≥95%), quercitrin (≥95%), rutin (≥94%), hesperidin (≥98%), luteolin-7-glucoside (≥98%), apigetrin (≥97%), astragalin (≥98%), apigenin (≥95%), luteolin (≥98%), DPPH (≥95%), and ascorbic acid (≥99%) were all obtained from Sigma-Aldrich (Darmstadt, Germany). Quercetin (≥95%) and protocatechuic acid (≥90%), as well as gallic acid (≥97%), were sourced from Merck (Darmstadt, Germany).

### 4.3. Growth Conditions and Treatment

For sterilization, seeds were first washed under running tap water and then immersed in 70% ethanol for 30 s. This was followed by surface sterilization with 5% sodium hypochlorite (NaClO) for 10 min. Finally, the seeds were rinsed three times with sterile distilled water for 5 min each to remove any residual NaClO. Sterilized seeds were germinated on PGR-free Murashige and Skoog medium (MS) [29].

After germination, plantlets were multiplied through subcultures on MS medium enriched with 0.5 mg L^−1^ BAP (6-benzylaminopurine) and 0.1 mg L^−1^ GA_3_ (gibberellic acid) The MS medium contained 3% sucrose and 0.6% agar, and the pH of the medium was adjusted to 5.8 [30]. The multiplication stage consisted of three consecutive subculture cycles, each lasting 4 weeks. Elongated shoots, approximately 2 cm in length with two leaves, were used as explants for subsequent experiments.

To evaluate the most effective cytokinin type and concentration for promoting plant growth, shoots were transferred to MS media containing different concentrations (0.1, 0.25, 0.5, and 1.0 mg L^−1^) of either BAP or zeatin (ZEA). PGR-free medium was used as control. Representative photographs of the shoot cultures from these treatment groups are provided in Figure S1.

All cultures, including those from seed germination, shoot multiplication, and subsequent cytokinin experimental treatments (50 mL Magenta™ vessels with ~30 mL MS medium), were maintained under controlled conditions in a growth room at 25 ± 2 °C, 16/8 h light/dark photoperiod, and 50 μmol m^−2^ s^−1^ light intensity.

At the end of the four-week growth period of the cytokinin treatment groups, the average dry weight, shoot number, and shoot length per explant of the shoots from the cytokinin treatment groups were recorded to monitor their growth performance and development. The dry weight (DW) of shoots was determined after drying the samples at 60 °C for 48 h until constant weight was reached.

### 4.4. Quantitative Evaluation of Phenolic Metabolites

The following extraction process was modified based on the protocol reported by Tusevski et al. (2017) [31]. After drying at room temperature, the shoots were weighed and subsequently powdered using a mortar. For extraction, 10 mL of 80% (*v*/*v*) methanol added to 0.2 g powdered material and mixture sonicated in an ice bath at 4 °C for 20 min (Sanyo Soniprep 150 (MSE, UK). This procedure was repeated three times, and the resulting supernatants were pooled. Following sonication, the methanolic extracts were centrifuged at 8000× *g* for 15 min using a Thermo Scientific Labofuge 200 (Thermo Fisher Scientific, Waltham, MA, USA). The resulting supernatant was passed through a 0.22 μm nylon syringe filter and kept at −20 °C in darkness until further analysis. For LC–MS/MS analysis, 0.2 g of each powdered shoot sample was extracted as described above. Three representative samples per treatment were analyzed. Before LC–MS/MS evaluation, a 20 μL aliquot of each extract was introduced into the system.

**Figure 1:**
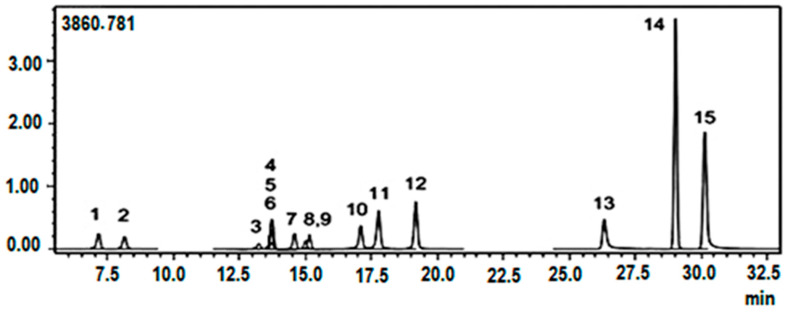
LC–MS/MS chromatograms of standard chemicals analyzed by LC–MS/MS method. 1 Protocatechuic acid. 2. Chlorogenic acid. 3 Luteolin-7-glucoside. 4 Rutin. 5 Hesperidin. 6 Hyperoside. 7 Apigetrin. 8 Quercitrine. 9 Astragalin. 10 Quercetin 11 Luteolin. 12 Apigenin. 13 Pseudohypericin. 14 Hyperforin. 15 Hypericin.

The qualitative and quantitative analysis of phytochemicals characteristic of *Hypericum* species was conducted using a validated LC–MS/MS method previously described by Akdeniz (2018) [32]. The LC–MS/MS system was operated in multiple reaction monitoring (MRM) mode, in which parent ions and their characteristic product ions were monitored for reliable identification and quantification of the analytes. This approach enables accurate detection of compounds even when chromatographic peaks are not completely separated in the chromatogram. The LC–MS/MS conditions were described in detail in our previous study [28]. Representative chromatograms of the standard compounds are shown in Figure 1. The comprehensive analytical parameters of the applied method are provided in Table S1, and detailed chromatograms of the samples are presented in Figure S2.

### 4.5. Determination of Total Phenolic Content (TPC)

Total phenolic content (TPC) of the extracts was determined using a modified colorimetric method based on the Folin–Ciocalteu assay, as described by Singleton et al. (1999) [33]. Briefly, 25 μL of the methanolic extract or standard solution was mixed with 1655 μL of distilled water. For the control group, 25 μL of 80% methanol was used instead of the plant extract. Subsequently, 100 μL of Folin–Ciocalteu reagent (Merck, Germany) was added to the mixture and vortexed thoroughly. After incubation for 8–10 min at room temperature, 300 μL of 7.5% (*w*/*v*) sodium carbonate solution was added.

The reaction mixture was then incubated for 90 min at room temperature in the dark. Absorbance was measured at 760 nm using a DU530 UV–Vis spectrophotometer (PG Instruments Limited, Lutterworth, UK). Gallic acid was used as the standard for the calibration curve, and results were expressed as milligrams of gallic acid equivalents per gram of dry weight (mg GAE g^−1^ DW).

### 4.6. Determination of Total Flavonoid Content (TFC)

The total flavonoid content (TFC) of the extracts was determined using a colorimetric assay based on a modified method described by Zhishen et al. (1999) [34]. Briefly, 100 μL of the methanolic extract was transferred into a test tube and mixed with 900 μL of distilled water. Subsequently, 60 μL of 5% (*w*/*v*) aqueous sodium nitrite (NaNO_2_) and 60 μL of 10% (*w*/*v*) aqueous aluminum chloride (AlCl_3_) were added sequentially and mixed thoroughly. Following this, 400 μL of 1 N sodium hydroxide (NaOH) and 450 μL of distilled water were added, and the reaction mixture was incubated for 40 min at room temperature in the dark.

After incubation, the absorbance was measured at 417 nm using a UV–Vis spectrophotometer. The results were expressed as milligrams of quercetin equivalents per gram of dry weight (mg QE g^−1^ DW).

### 4.7. Determination of DPPH Radical Scavenging Activity

The free radical scavenging activity of the extracts was evaluated using the 2,2-diphenyl-1-picrylhydrazyl (DPPH) assay according to the method described by Brand-Williams et al. (1995) [35]. Briefly, 500 μL of the plant extract was mixed with 500 μL of freshly prepared DPPH solution. The reaction mixture was incubated for 20 min at room temperature in the dark.

After incubation, the decrease in absorbance was measured at 517 nm using a UV–Vis spectrophotometer. Ascorbic acid was used as the reference standard. Standard solutions were prepared at concentrations of 31–350 µg/mL. The antioxidant activity of the extracts was calculated from this curve and expressed as milligrams of ascorbic acid equivalents per gram of dry weight (mg ASA/g DW).

### 4.8. Statistical Analysis

For growth parameters, including shoot number, shoot length, and dry weight per explant, each cytokinin treatment was applied to five independent culture vessels (n = 5), with five explants per vessel. Measurements from the five explants in each vessel were averaged, and the vessel mean was used as a biological replicate for statistical analysis. For biochemical analyses, including phenolic compound contents, total phenolic content (TPC), total flavonoid content (TFC), and DPPH radical scavenging activity, three independent vessels per treatment were sampled and pooled for extraction, and each extract was analyzed in triplicate. Statistical differences among treatments were evaluated using one-way analysis of variance (ANOVA), followed by Duncan’s multiple range test at *p* < 0.05, using using IBM SPSS Statistics 31.0 software (IBM Corp., Armonk, NY, USA) [36].

## 5. Conclusions

Phenolic acids and flavonoids, such as protocatechuic acid, chlorogenic acid, quercetin, and hyperforin, contribute significantly to the broad biological activity of the genus. These bioactive constituents highlight the importance of developing efficient strategies for the sustainable production of plant-derived compounds.

Our study demonstrated that cytokinin supplementation markedly influences both morphogenesis and secondary metabolite accumulation in *H. amblysepalum* shoot cultures. While BAP was more effective in promoting shoot proliferation and biomass production, ZEA preferentially enhanced the accumulation of phenolic compounds, particularly hypericin and pseudohypericin. This cytokinin-specific response suggests that BAP primarily stimulates rapid cell division, whereas ZEA supports a more balanced growth pattern favoring secondary metabolite biosynthesis.

In the present study, LC–MS/MS profiling and spectrophotometric analyses demonstrated that treatments resulting in higher total phenolic and flavonoid contents also exhibited increased DPPH radical scavenging activity in *H. amblysepalum* shoot cultures.

Overall, these findings indicate that cytokinin-regulated shoot cultures can modulate both morphogenesis and secondary metabolite profiles. While total phenolic content was highest in PGR-free cultures, specific cytokinin treatments, particularly ZEA, enhanced the accumulation of certain bioactive compounds, suggesting that these cultures represent a controlled biotechnological system for targeted metabolite production.

## Figures and Tables

**Table 1:** Effect of cytokinins BAP and ZEA on the growth of *Hypericum amblysepalum* shoots.

PGR Concentration (mg L^−1^)	Average Dry Weight (per Explant)		Average Shoot Numbers (per Explant)		Average Shoot Lenght (cm)	
	BAP	ZEA	BAP	ZEA	BAP	ZEA
0.0	5.27 ± 0.35 ^b^	5.27 ± 0.35 ^b^	1.0 ± 0.00 ^c^	1.0 ± 0.00 ^a^	4.8 ± 0.78 ^a^	4.8 ± 0.78 ^a^
0.1	26.5 ± 2.15 ^a^	16.9 ± 1.39 ^a^	11.29 ± 4.48 ^a^	2.76 ± 0.75 ^a^	2.1 ± 0.66 ^b^	3.2 ± 0.88 ^b^
0.25	23.4 ± 4.57 ^a^	13.0 ± 1.50 ^a^	9.07 ± 3.25 ^b^	2.33 ± 0.90 ^a^	1.7 ± 0.48 ^b^	2.9 ± 0.77 ^b^
0.5	19.5 ± 3.24 ^a^	19.5 ± 2.15 ^a^	7.00 ± 2.29 ^b^	3.11 ± 0.92 ^a^	1.8 ± 0.78 ^b^	3.4 ± 0.75 ^b^
1.0	20.2 ± 3.95 ^a^	17.5 ± 2.23 ^a^	6.73 ± 2.21 ^b^	2.88 ± 1.31 ^a^	1.03 ± 0.39 ^b^	3.0 ± 1.2 ^b^

Values are presented as mean ± SE (n = 5). Means followed by the same letter within a column are not significantly different at *p* ≤ 0.05 according to Duncan’s multiple range test.

**Table 2:** Effect of cytokinins BAP and ZEA on the phenolic compound contents of *Hypericum amblysepalum* shoot cultures (μg/g extract).

Phenolic			Cytokinin Treatment (mg L^−1^)							
Compounds	RT	Control	0.1 BAP	0.25 BAP	0.5 BAP	1.0 BAP	0.1 ZEA	0.25 ZEA	0.5 ZEA	1.0 ZEA
Hypericin	30.18	6.70 ± 1.12 ^e^	28.80 ± 0.54 ^bc^	18.65 ± 0.35 ^c^	17.80 ± 0.33 ^c^	11.90 ± 0.22 ^d^	52.60 ± 0.99 ^a^	33.70 ± 0.63 ^b^	51.50 ± 0.97 ^a^	31.90 ± 1.33 ^b^
Pseudohypericin	26.34	47.21 ± 0.81 ^e^	149.90 ± 2.57 ^ab^	92.80 ± 1.5 ^c^	81.90 ± 1.40 ^c^	63.50 ± 1.09 ^d^	169.90 ± 2.92 ^a^	115.80 ± 1.99 ^b^	139.70 ± 2.40 ^ab^	123.70 ± 2.12 ^b^
Hyperforin	28.97	N.D.	N.D.	N.D.	N.D.	N.D.	3.100 ± 0.12	0.12 ± 0.005	N.D.	4.60 ± 0.19
Rutin	13.67	156.20 ± 2.12 ^c^	193.70 ± 2.63 ^b^	134.50 ± 1.82 ^d^	150.10 ± 2.04 ^cd^	128.90 ± 1.7 ^d^	261.50 ± 3.55 ^a^	150.40 ± 2.04 ^cd^	183.30 ± 2.49 ^bc^	162.80 ± 2.21 ^c^
Hesperidin	13.68	97.60 ± 1.58 ^b^	103.02 ± 1.66 ^b^	72.20 ± 1.16 ^c^	80.30 ± 1.30 ^c^	68.20 ± 1.10 ^c^	123.35 ± 1.99 ^a^	76.40 ± 1.23 ^c^	94.40 ± 1.52 ^b^	84.20 ± 1.36 ^c^
Hyperoside	13.69	3146.5 ± 39.6 ^b^	2067.5 ± 26.05 ^d^	1539.4 ± 19.3 ^e^	1621.8 ± 20.43 ^e^	1476.7 ± 18.60 ^e^	3744.9 ± 47.18 ^a^	2598.4 ± 32.74 ^c^	2960.3 ± 37.30 ^b^	2897.2 ± 36.50 ^b^
Quercetin	17.10	19.80 ± 1.13 ^b^	38.40 ± 2.20 ^a^	30.40 ± 1.74 ^ab^	51.10 ± 2.92 ^a^	27.55 ± 1.57 ^ab^	45.80 ± 2.62 ^a^	27.90 ± 1.59 ^ab^	32.30 ± 1.85 ^ab^	32.30 ± 1.85 ^ab^
Quercitrin	14.98	128.00 ± 1.7 ^a^	20.80 ± 0.27 ^e^	25.80 ± 0.34 ^d^	16.90 ± 0.22 ^f^	11.35 ± 0.37 ^g^	56.95 ± 0.75 ^b^	42.60 ± 0.56 ^c^	47.80 ± 0.63 ^ac^	47.30 ± 0.62 ^c^
Luteolin	17.78	3.01 ± 0.05 ^b^	1.10 ± 0.02 ^e^	0.90 ± 0.016 ^f^	0.85 ± 0.015 ^f^	1.50 ± 0.028 ^d^	4.20 ± 0.07 ^a^	1.50 ± 0.02 ^d^	1.60 ± 0.03 ^d^	2.50 ± 0.04 ^c^
Astragalin	15.13	23.30 ± 0.35 ^a^	15.06 ± 0.23 ^c^	10.30 ± 0.15 ^d^	7.70 ± 0.11 ^e^	7.03 ± 0.10 ^e^	20.30 ± 0.31 ^b^	16.70 ± 0.25 ^c^	16.40 ± 0.25 ^c^	16.90 ± 0.25 ^c^
Apigenin	19.20	0.25 ± 0.004 ^e^	0.94 ± 0.017 ^b^	0.59 ± 0.010 ^c^	0.93 ± 0.016 ^b^	0.40 ± 0.007 ^d^	0.20 ± 0.003 ^e^	0.10 ± 0.001 ^f^	0.68 ± 0.012 ^c^	0.10 ± 0.001 ^f^
Luteolin-7-glucoside	13.20	4.65 ± 0.03 ^b^	3.60 ± 0.03 ^c^	2.70 ± 0.02 ^d^	2.50 ± 0.02 ^e^	3.60 ± 0.03 ^c^	6.04 ± 0.05 ^a^	4.60 ± 0.039 ^b^	3.65 ± 0.031 ^c^	4.35 ± 0.037 ^b^
Apigetrin	14.54	N.D.	N.D.	N.D.	N.D.	N.D.	N.D.	N.D.	N.D.	N.D.
Protocatechuic acid	7.00	12.62 ± 0.27 ^d^	17.90 ± 0.38 ^ab^	10.90 ± 0.23 ^e^	20.35 ± 0.43 ^a^	14.80 ± 0.31 ^c^	18.20 ± 0.39 ^a^	12.35 ± 0.26 ^d^	18.01 ± 0.30 ^a^	18.10 ± 0.38 ^a^
Chlorogenic acid	8.03	29.90 ± 0.89 ^d^	27.01 ± 0.80 ^d^	23.90 ± 0.71 ^e^	50.80 ± 1.51 ^b^	22.40 ± 0.66 ^e^	66.30 ± 1.98 ^a^	51.90 ± 1.55 ^b^	44.02 ± 1.31 ^c^	44.20 ± 1.32 ^c^

Values are presented as mean ± SE (n = 3). Means followed by the same letter within a column are not significantly different at *p* ≤ 0.05 according to Duncan’s multiple range test. N.D.: not detected; RT: retention time (min).

**Table 3:** Effect of BAP and ZEA treatments on total phenolic content (TPC), total flavonoid content (TFC) and DPPH radical scavenging activity of *Hypericum amblysepalum* shoot cultures.

Cytokinin Treatment	TPC (mg GAE g^−1^ DW)	TFC (mg QE g^−1^ DW)	Antioxidant Activity (mg ASA g^−1^ DW)
PGR-free	18.11 ± 0.58 ^a^	43.79 ± 0.98 ^a^	18.2 ± 0.001 ^a^
0.1 BAP	9.31 ± 0.66 ^bc^	21.11 ± 1.78 ^c^	17.2 ± 0.002 ^a^
0.25 BAP	8.64 ± 2.63 ^bc^	13.93 ± 0.84 ^c^	17.45 ± 0.017 ^a^
0.5 BAP	9.02 ± 1.22 ^bc^	21.30 ± 2.13 ^c^	17.4 ± 0.015 ^a^
1.0 BAP	6.47 ± 1.02 ^c^	13.27 ± 1.16 ^c^	17.7 ± 0.006 ^a^
0.1 ZEA	12.48 ± 0.46 ^b^	38.37 ± 0.58 ^ab^	18.0 ± 0.002 ^a^
0.25 ZEA	10.59 ± 1.29 ^b^	35.76 ± 2.62 ^ab^	18.15 ± 0.011 ^a^
0.5 ZEA	11.79 ± 1.14 ^b^	37.13 ± 1.11 ^ab^	17.75 ± 0.015 ^a^
1.0 ZEA	8.44 ± 0.34 ^bc^	21.86 ± 2.40 ^c^	18.65 ± 0.010 ^a^

Values are expressed as mean ± SD (n = 3). Different letters within the same column indicate statistically significant differences (*p* < 0.05).

## Data Availability

The original contributions presented in this study are included in the article/Supplementary Material. Further inquiries can be directed to the corresponding author.

## Supplementary Materials

The following supporting information can be downloaded at https://www.mdpi.com/article/10.3390/plants15071017/s1, Figure S1: In vitro shoot cultures of *H. amblysepalum* grown on MS media supplemented with different cytokinin treatments. Figure S2: Representative LC–MS/MS chromatograms of control and cytokinin-treated *H. amblysepalum* shoot cultures. Table S1: The comprehensive analytical parameters of the applied method (see Ref. [37]).
