# Supplementary material for: Modulation of Plant-Derived Bioactive Phenolic Compounds by Cytokinins in Hypericum amblysepalum Shoot Cultures
Source: Plants (Basel). 2026 Mar 26;15(7):1017. doi: 10.3390/plants15071017 (PMC13074684; doi:10.3390/plants15071017)
Supplement: Supplementary file 1 [file plants-15-01017-s001.zip › Supplemantary material 3- Figure S2 The LC–MS MS chromatograms of Sample_compressed.pdf]

Modulation of Plant-Derived Bioactive Phenolic Compounds by Cytokinins in *Hypericum amblysepalum* Shoot Cultures

Hilal SURMUŞ ASAN

Department of Biology, Faculty of Science, Dicle Universty, 21280, Diyarbakır, TURKEY

Figure S2: Representative LC–MS/MS chromatograms of standarts, control and cytokinin-treated *Hypericum amblysepalum* shoot cultures.

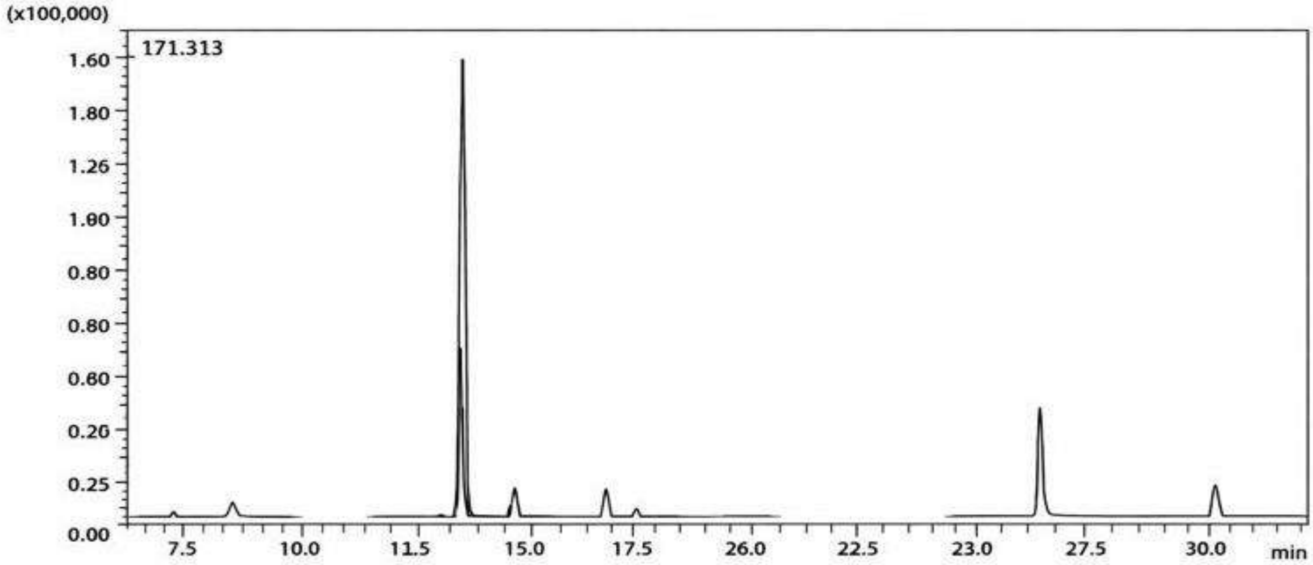

CONTROL

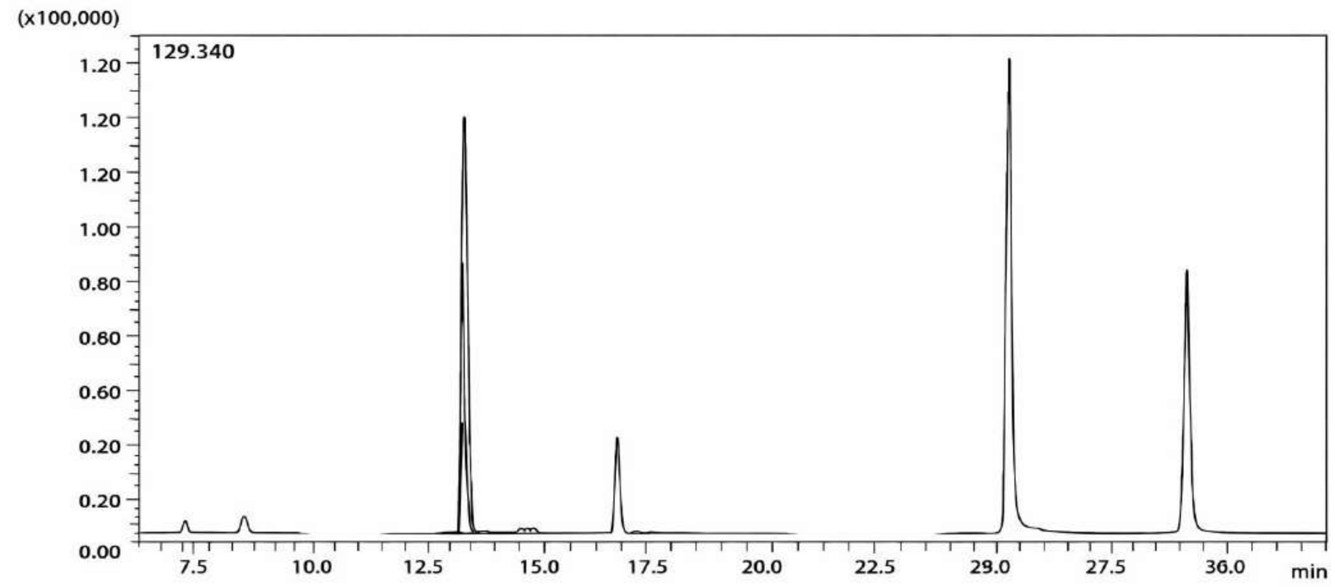

0.1 mg/L BAP

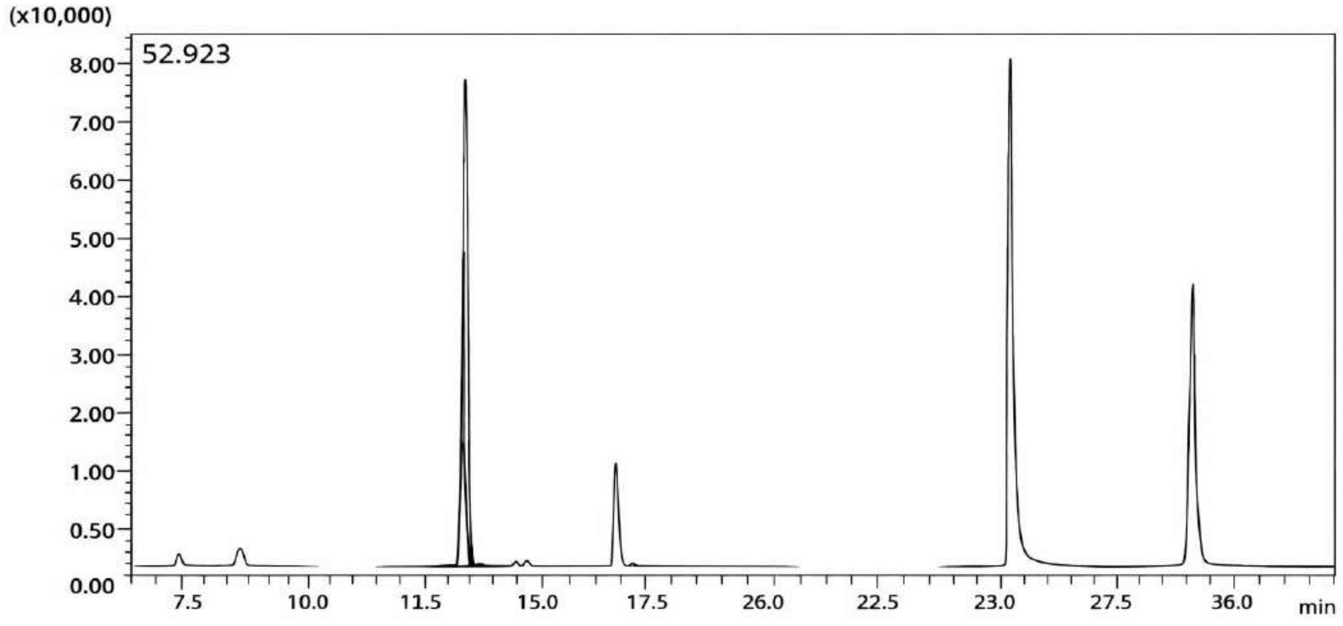

0.25 mg/L BAP

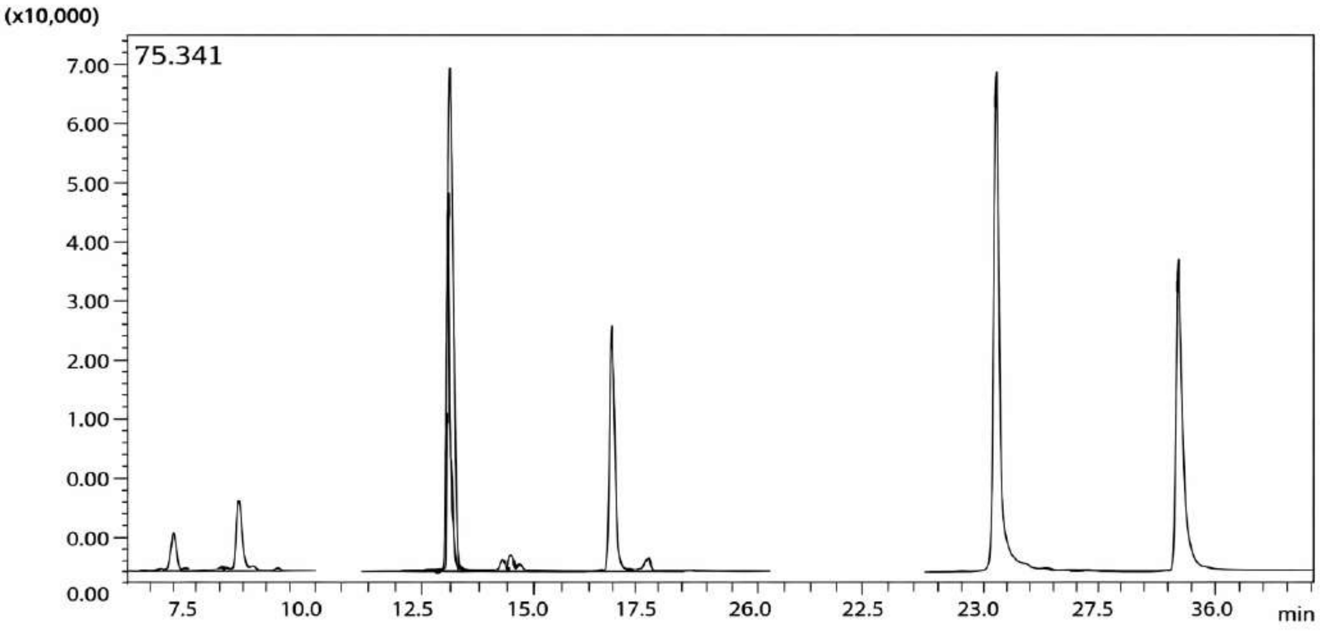

0.5 mg/L BAP

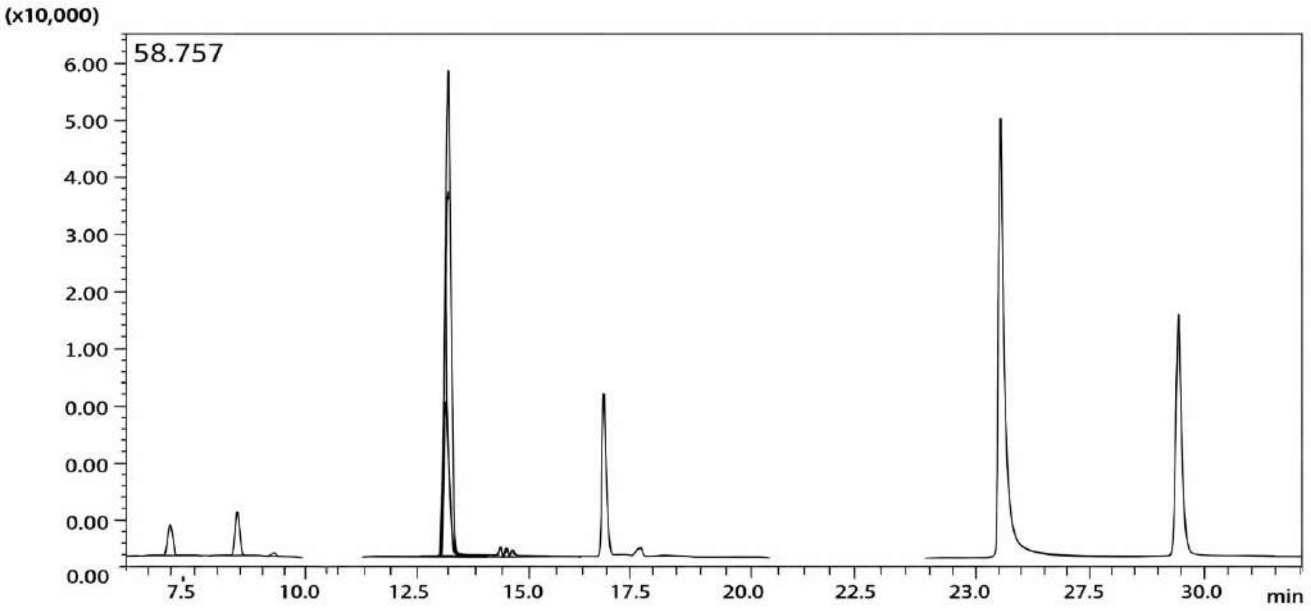

1.0 mg/L BAP

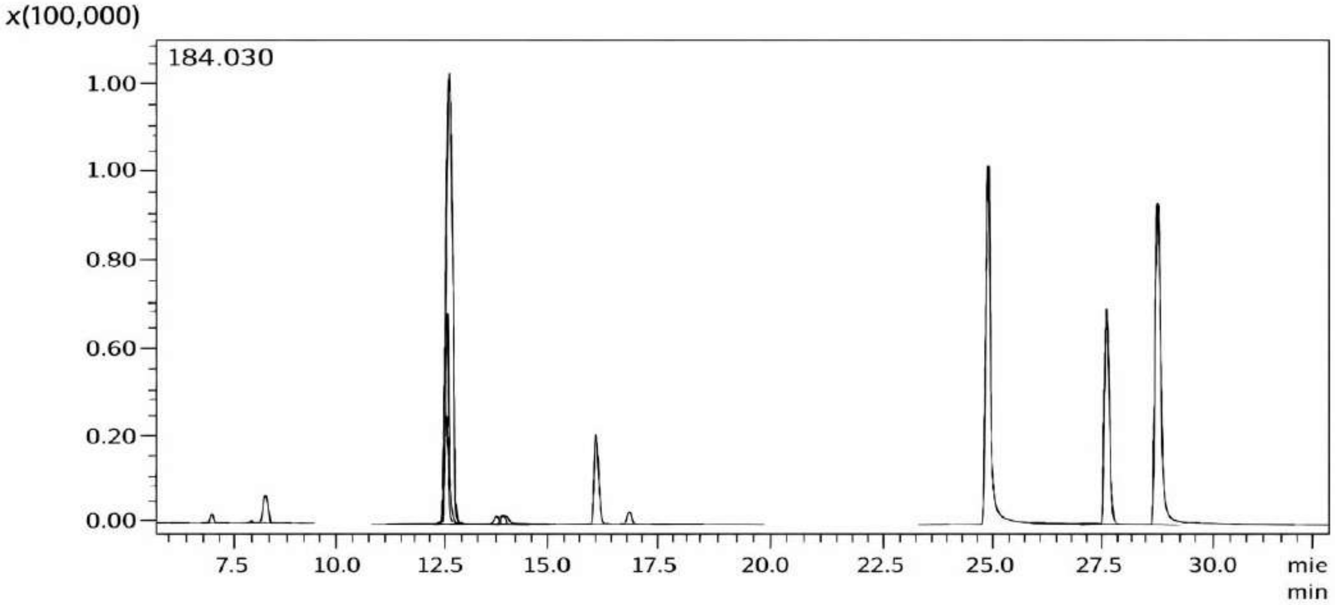

0.1 mg/L ZEA

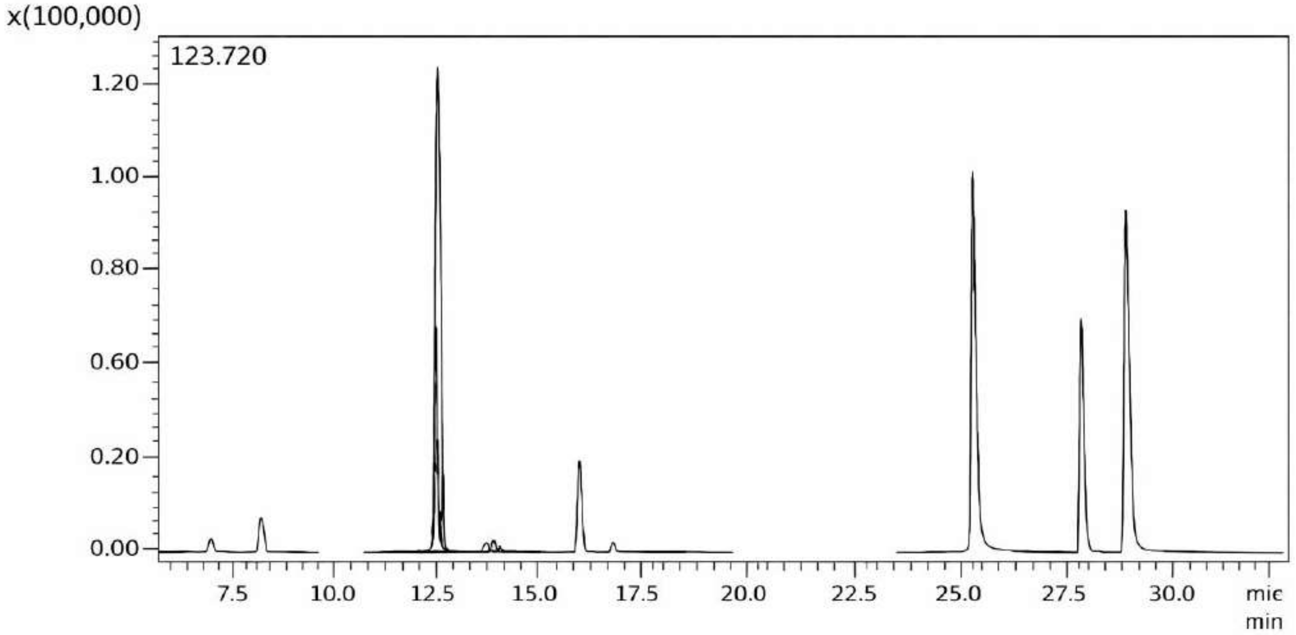

0.25 mg/L ZEA

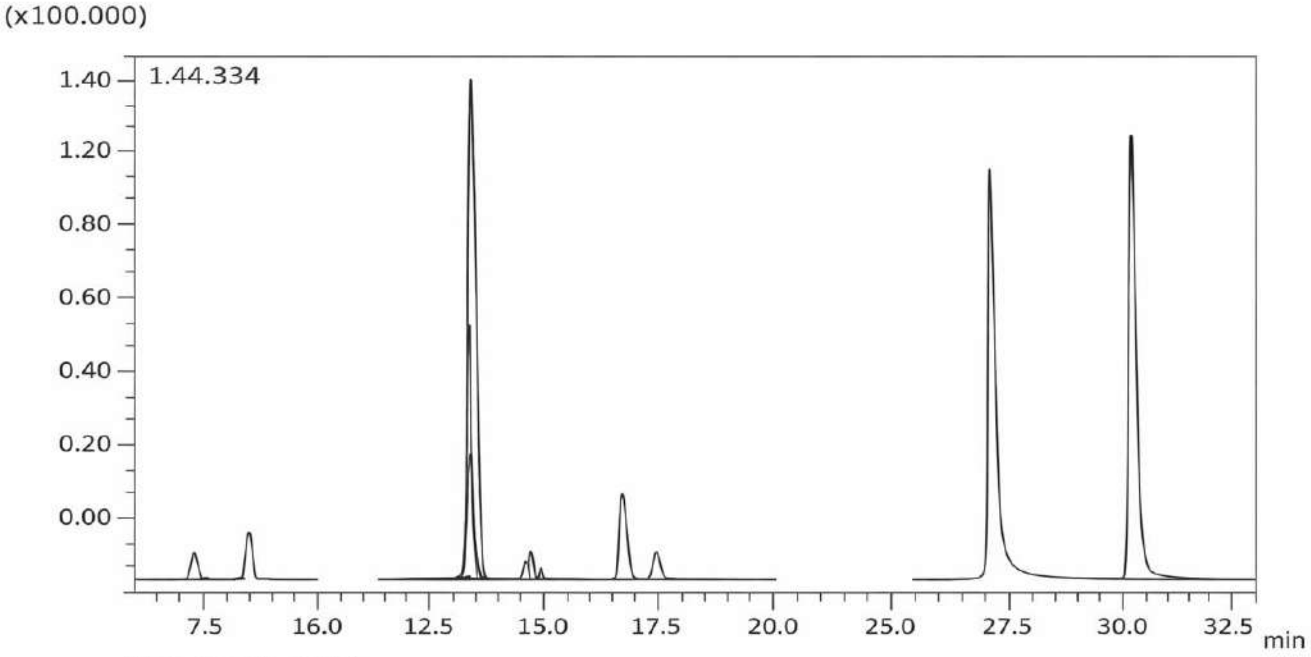

0.5 mg/L ZEA

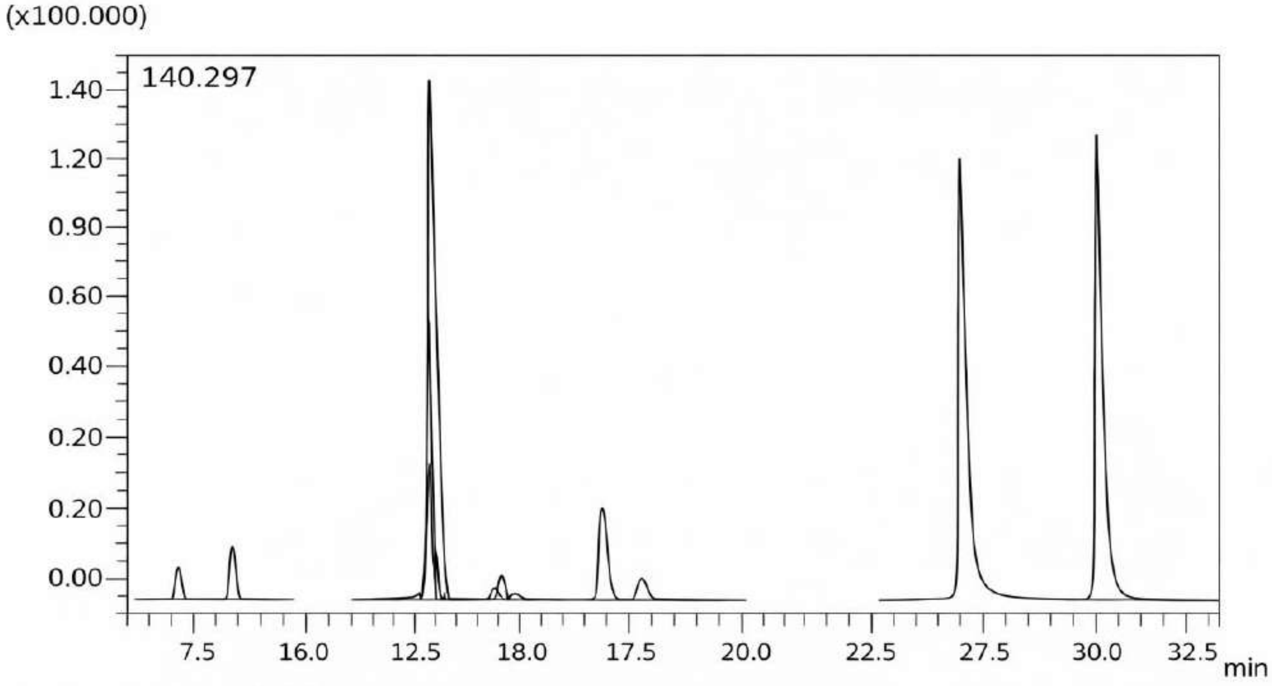

1.0 mg/L ZEA
