# Supplementary material for: Modulation of Plant-Derived Bioactive Phenolic Compounds by Cytokinins in Hypericum amblysepalum Shoot Cultures
Source: Plants (Basel). 2026 Mar 26;15(7):1017. doi: 10.3390/plants15071017 (PMC13074684; doi:10.3390/plants15071017)
Supplement: Supplementary file 1 [file plants-15-01017-s001.zip › Supplementary material 2-Table S1 Analytical parameters of LC–MS MS method_compressed.pdf]

Modulation of Plant-Derived Bioactive Phenolic Compounds by Cytokinins in Hypericum amblysepalum Shoot Cultures

Hilal SURMUŞ ASAN*
*Department of Biology, Faculty of Science, Dicle Universty, Diyarbakır, TURKEY
*Corresponding author: hilalsuran@gmail.com,

Table S1. Analytical parameters of LC–MS/MS method for 15 phytochemicals (Akdeniz 2020)

								(% RSD[d])				Recovery (%)		
No	Analytes	Mother ion (m/z)[a]	RT[b]	Fragment ions	Ion mod	Equation	R^{2c}	Inter day	Intra day	Linear Range (µg/L)	LOD/LOQ (µg/L)[e]	Inter day	Intra day	U[f]
1.	Protocatechuic acid	153.4	7.00	109.1-108.0	Neg	y=590.460x+120,226	0.9909	0.60	0.60	100-3200	4.26/5.32	100.96	99.88	0.0215
2.	Chlorogenic acid	353.3	8.03	191.2-85.0	Neg	y=697.935x+87,418.5	0.9910	0.74	0.55	75-2400	2.44/3.36	99.41	99.99	0.0299
3.	Luteolin-7-glucoside	447.0	13.20	285.1-284.1	Neg	y=215.412x+36,852.1	0.9939	0.52	0.37	75-2400	2.30/3.02	100.14	100.72	0.0086
4.	Rutin	609.1	13.67	300.1-301.1	Neg	y=469.333x+30,144.8	0.9902	0.63	0.70	100-3200	1.283/1.90	100.49	100.37	0.0136
5.	Hesperidin	611.1	13.68	303.0-449.3	Pos	y=2539.52x+123.981	0.9942	0.81	0.73	50-1600	0.96/1.44	100.53	99.94	0.0162
6.	Hyperoside	463.0	13.69	301.1-271.0	Neg	y=185.593x+8126.67	0.9905	0.74	0.56	100-3200	5.48/6.50	100.39	100.15	0.0126
7.	Apigetrin	431.0	14.54	268.1-269.1	Neg	y=1052.01x+146,897	0.9902	0.47	0.67	50-1600	1.23/1.75	100.60	100.47	0.0132
8.	Quercitrin	447.0	14.98	300.0-301.1	Neg	y=175.298x+33,626.6	0.9918	0.79	0.63	100-3200	1.051/1.65	99.99	100.02	0.0133
9.	Astragalin	447.0	15.13	284.1-227.1	Neg	y=329.506x+44,598.6	0.9900	0.86	0.77	100-3200	5.52/6.77	100.02	100.17	0.0153
10.	Quercetin	301.2	17.10	151.1-179.1	Neg	y=1826.89x-146948	0.9962	1.17	2.27	50-1600	1.25/1.81	100.10	100.12	0.0573
11.	Luteolin	285.2	17.78	133.1-151.0	Neg	y=3166.03x+495,252	0.9901	1.19	0.79	50-1600	0.61/0.87	99.61	100.07	0.0188
12.	Apigenin	269.2	19.20	117.0-151.1	Neg	y=3115.89x+483,037	0.9910	0.87	0.90	50-1600	0.32/0.52	99.85	100.22	0.0181
13.	Pseudohypericin	519.0	26.34	487.1-475.1	Neg	y=2548. 96x+468,900	0.9908	0.61	0.89	50-1600	2.15/2.55	100.33	100.34	0.0172
14.	Hyperforin	535.3	28.97	383.3-315.2	Neg	y=44,260. 6x+203,394	0.9901	0.18	1.64	10-320	0.32/0.51	100.76	100.61	0.0418
15.	Hypericin	503.0	30.18	405.1-433.1	Neg	y=7676.03x+605,593	0.9925	0.93	0.95	50-1600	1.27/1.88	101.04	100.34	0.0189

[a] Mother ion (m/z): molecular ions of the standard compounds (m/z ratio), [b] RT: retention time (min), [c]R^2: coefficient of determination, [d] RSD: relative standard deviation, [e] LOD/LOQ (µg/L): limit of detection/quantification, [f] U: relative uncertainty at 95% confidence level (k = 2)

* Akdeniz, M., Yilmaz, M. A., Ertas, A., Yener, I., Firat, M., Aydin, F., & Kolak, U. (2020). Method validation of 15 phytochemicals in Hypericum lysimachioides var. spathulatum by LC–MS/MS, and fatty acid, essential oil, and aroma profiles with biological activities. Journal of Food Measurement and Characterization, 14(6), 3194-3205.
