# Supplementary material for: Modulation of Plant-Derived Bioactive Phenolic Compounds by Cytokinins in Hypericum amblysepalum Shoot Cultures
Source: Plants (Basel). 2026 Mar 26;15(7):1017. doi: 10.3390/plants15071017 (PMC13074684; doi:10.3390/plants15071017)
Supplement: Supplementary file 1 [file plants-15-01017-s001.zip › Supplementary Material 1-Figure S1_compressed.pdf]

Modulation of Plant-Derived Bioactive Phenolic Compounds by Cytokinins in *Hypericum amblysepalum* Shoot Cultures

Hilal SURMUŞ ASAN

Department of Biology, Faculty of Science, Dicle Universty, 21280, Diyarbakır, TURKEY

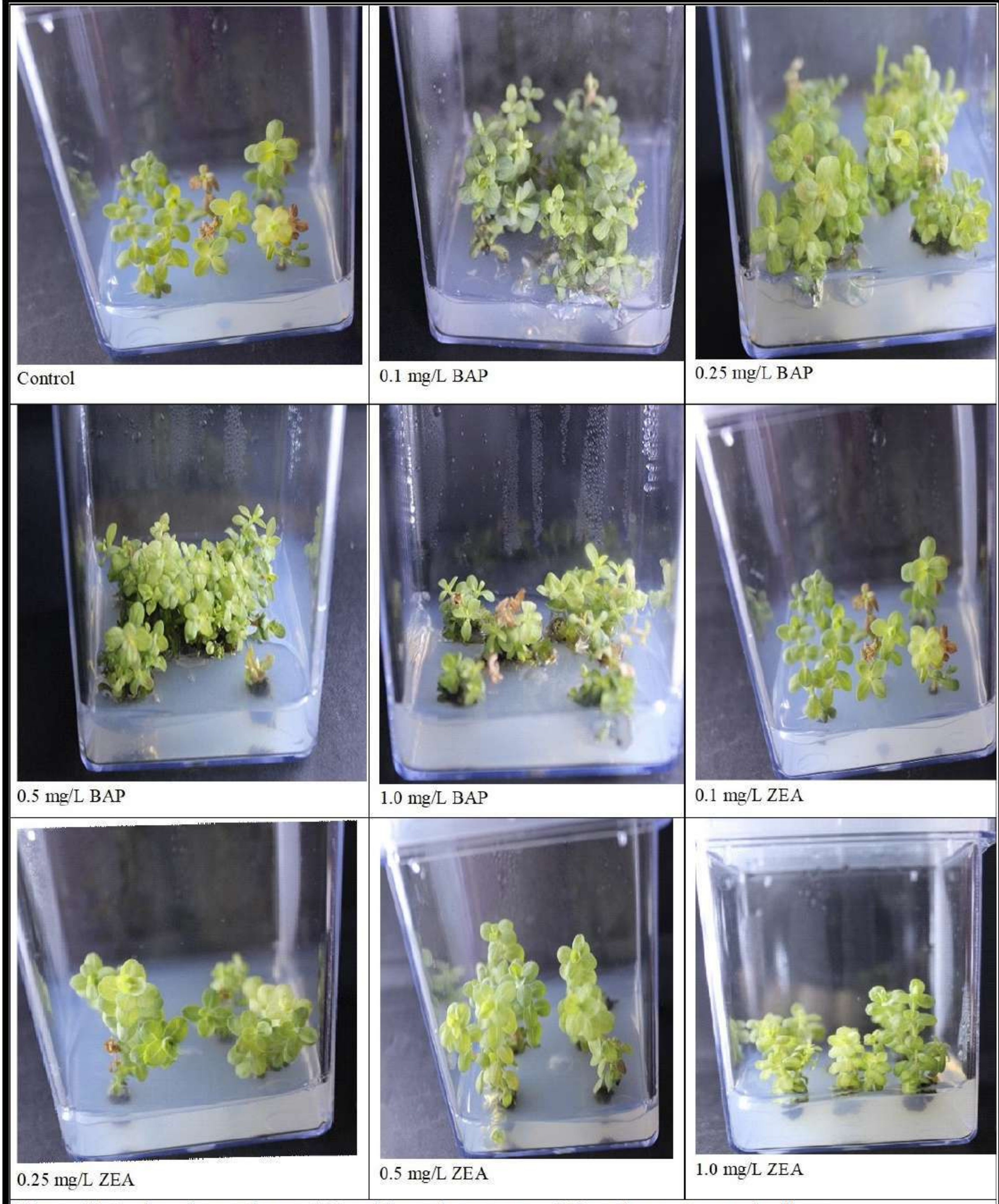

Figure S1: In vitro shoot cultures of *H. amblysepalum* grown on MS media supplemented with different cytokinin treatments.
